# Association between maternal and paternal mental illness and risk of injuries in children and adolescents: nationwide register based cohort study in Sweden

**DOI:** 10.1136/bmj.m853

**Published:** 2020-04-08

**Authors:** Alicia Nevriana, Matthias Pierce, Christina Dalman, Susanne Wicks, Marie Hasselberg, Holly Hope, Kathryn M Abel, Kyriaki Kosidou

**Affiliations:** 1 Department of Global Public Health, Karolinska Institutet, 17177 Stockholm, Sweden; 2 Centre for Women’s Mental Health, Division of Psychology and Mental Health, Faculty of Biology, Medicine and Health Sciences, University of Manchester, Manchester, UK; 3 Center for Epidemiology and Community Medicine, Stockholm Region, Stockholm, Sweden; 4 Greater Manchester Mental Health NHS Foundation Trust, Manchester, UK

## Abstract

**Objective:**

To determine the association between parental mental illness and the risk of injuries among offspring.

**Design:**

Retrospective cohort study.

**Setting:**

Swedish population based registers.

**Participants:**

1 542 000 children born in 1996-2011 linked to 893 334 mothers and 873 935 fathers.

**Exposures:**

Maternal or paternal mental illness (non-affective psychosis, affective psychosis, alcohol or drug misuse, mood disorders, anxiety and stress related disorders, eating disorders, personality disorders) identified through linkage to inpatient or outpatient healthcare registers.

**Main outcome measures:**

Risk of injuries (transport injury, fall, burn, drowning and suffocation, poisoning, violence) at ages 0-1, 2-5, 6-9, 10-12, and 13-17 years, comparing children of parents with mental illness and children of parents without mental illness, calculated as the rate difference and rate ratio adjusted for confounders.

**Results:**

Children with parental mental illness contributed to 201 670.5 person years of follow-up, while children without parental mental illness contributed to 2 434 161.5 person years. Children of parents with mental illness had higher rates of injuries than children of parents without mental illness (for any injury at age 0-1, these children had an additional 2088 injuries per 100 000 person years; number of injuries for children with and without parental mental illness was 10 235 and 72 723, respectively). At age 0-1, the rate differences ranged from 18 additional transport injuries to 1716 additional fall injuries per 100 000 person years among children with parental mental illness compared with children without parental mental illness. A higher adjusted rate ratio for injuries was observed from birth through adolescence and the risk was highest during the first year of life (adjusted rate ratio at age 0-1 for the overall association between any parental mental illness that has been recorded in the registers and injuries 1.30, 95% confidence interval 1.26 to 1.33). Adjusted rate ratios at age 0-1 ranged from 1.28 (1.24 to 1.32) for fall injuries to 3.54 (2.28 to 5.48) for violence related injuries. Common and serious maternal and paternal mental illness was associated with increased risk of injuries in children, and estimates were slightly higher for common mental disorders.

**Conclusions:**

Parental mental illness is associated with increased risk of injuries among offspring, particularly during the first years of the child’s life. Efforts to increase access to parental support for parents with mental illness, and to recognise and treat perinatal mental morbidity in parents in secondary care might prevent child injury.

## Introduction

A recent national survey in the United States estimated that approximately 18% of all parents had some type of mental illness during the past year, and about 4% had serious mental illness.^1^ A study in the United Kingdom that used primary care data reported that nearly a quarter of children are exposed to maternal mental illness.^2^ In Sweden, we used secondary care data to estimate the prevalence of children and adolescents with parental mental illness (CAPRI) to be around 7% at 0-3 years, rising to nearly 11% by 15-17 years (Pierce et al, unpublished data, 2020). Importantly, mental illness contributes to the largest share of years lived with disability (around 20-30%) among young adults aged 15-29,^3^ and coincides with the most common age at which women and men first become parents.^4^ ^5^

For professionals working to support families with parental mental illness, safeguarding of children has focused on neglect and maltreatment; less focus has been put on preventing accidents and injuries, or improving the social factors that might influence outcomes in these children.^6^ ^7^ However, injuries for which evidence of successful preventive measures exists^8^ are still one of the leading causes of preventable disability and premature death in children and adolescents globally.^9^ Among 0-17 year olds in Sweden, injuries represent the third commonest cause of death, while the commonest cause of admission to hospital in young people is fall injuries followed by transport injuries.^10^

With the exception of two studies,^11^ ^12^ the literature examining associations between parental mental illness^12^ ^13^ ^14^ ^15^ ^16^ ^17^ ^18^ ^19^ ^20^ ^21^ ^22^ ^23^ ^24^ or mental illness in adults living with children^11^ and childhood injuries has only reported risks with common mental disorders such as depression^13^ ^14^ ^16^ ^17^ ^18^ ^19^ ^20^ ^21^ ^22^ ^23^ and substance use disorders among parents.^15^ ^19^ ^24^ Many studies evaluated mental illness through self-report rather than clinical diagnosis,^13^ ^17^ ^18^ ^20^ ^21^ ^22^ ^23^ and most assessed the effect of maternal mental illness only,^12^ ^13^ ^14^ ^16^ ^17^ ^18^ ^19^ ^20^ ^22^ ^23^ or did not specify which parent was assessed.^24^ Only two studies have examined the effect of both maternal and paternal mental illness on risk of injuries in children.^15^ ^21^ One study reported higher injury risk for children exposed to maternal substance misuse than paternal substance misuse^15^; another study with a small sample size (n=584) reported similar increased injury risk with maternal and paternal depression.^21^ Three previous studies have reported risks for specific types of injuries, mainly burn and poisoning, but also fall and drowning.^16^ ^17^ ^19^ However, all of these studies only assessed risks for maternal depression among infants and preschool children^16^ ^17^ ^19^; one study used self-reported information on injuries^17^; and none of them assessed risks for all types of childhood injuries. Finally, most previous studies focus on infant and preschool children without examining risks across childhood into adolescence, which is a time of heightened injury risk, especially for boys.^25^ ^26^

We address these important gaps in the available information using Sweden’s high quality population registers. Our aim was to detail associations between all types of maternal and paternal mental illness and risk of different types of injuries among offspring from birth through adolescence. We hypothesised that injury risk would be greatest in the first years of life when children are very dependent on their parents, and higher for maternal mental illness exposure because the mother is often the primary care giver. We also hypothesised that the risk might be higher for serious mental illness, such as psychosis, than more common mental disorders, such as depression and anxiety.

## Methods

### Study design and settings

We conducted a retrospective cohort study that included data from high quality, nationwide, and longitudinal health and administrative registers. We used Psychiatry Sweden to obtain data, a register linkage especially designed to study the occurrence, causes, and consequences of mental illness. The registers in Psychiatry Sweden were linked using the unique personal identification number assigned to each Swedish resident at birth or upon migration to Sweden.^27^ The following registers were used in this study:

- The Total Population Register, which contains demographic data on Swedish residents, with 100% coverage on vital status and more than 90% coverage on migration information.^28^
- The Multi-Generation Register, which links people to their biological or adoptive parents with almost complete coverage.^29^ ^30^
- The National Patient Register, which includes records of all inpatient care in Sweden, with complete coverage for psychiatric disorders since 1973 and somatic disorders since 1987; and records on specialised outpatient care visits since 2001 (with near complete coverage since 2006).^31^ ^32^ Healthcare is universal in Sweden and healthcare visits are free of charge for children; adults are only required to pay a minimum amount annually (eg, for outpatient care: Kr1150; £96; €103; $111).^33^ Healthcare visits recorded in the National Patient Register include information on the patient (sex, age, place of residence), the care giver (hospital or department), date of admission and discharge (inpatient), date of visit (outpatient), ICD (international classification of disease) based diagnoses and external codes (for injury).^32^ Missing information on the main diagnosis was estimated to be around 0.9% for hospitalisations and around 3.2% for outpatient visits, as of 2018.^34^
- The Longitudinal Integration Database for Health Insurance and Labour Market Studies, which contains data on socioeconomic variables for people aged 16 years and older since 1990, with estimated completeness of more than 95%.^35^

These registers have been used extensively for research and have been previously validated by using external sources of information.^28^ ^30^ ^32^ ^35^ ^36^ ^37^ ^38^ ^39^

### Study population

**Fig 1:**
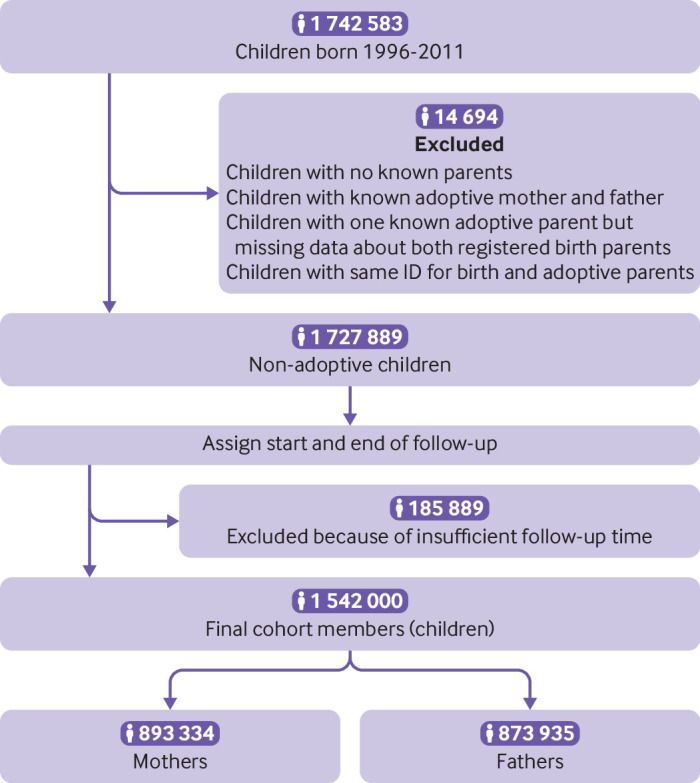
Flowchart of the analytical sample

The study population was defined as all children living in Sweden who were born between 1996 and 2011, who were identified through the Total Population Register (n=1 742 583). These children were linked to their registered birth parents by using the Multi-Generation Register. We excluded children with no known parents; those with known adoptive parents (mother and father); children with one known adoptive parent but with missing information about both registered birth parents; and children with the same ID for birth and adoptive parents (n=14 694). Children were followed from their date of birth or five years after their parents’ immigration date to Sweden, whichever was later. Children were followed until the date of either parent’s emigration from Sweden, the child’s death, death of either parent, age 18, or 31 December 2016, whichever was earliest. Children’s follow-up was split into the predefined developmental age periods: infancy (0-1 year), preschool (2-5 years), primary school (6-9 years), lower secondary school (10-12 years), and adolescence (13-17 years). The final cohort available for analysis consisted of 1 542 000 children linked to 893 334 mothers and 873 935 fathers (fig 1), which corresponded to an average of 1.7 children (standard deviation 0.8) per parent.

### Parental mental illness

For each developmental period, we considered children to be exposed to maternal or paternal mental illness if their mother or father was diagnosed with a mental illness within inpatient or outpatient mental healthcare (or both). This information was recorded on the National Patient Register during that specific developmental period or in the previous five years (see supplementary fig 1). We chose this approach because mental illnesses are chronic or tend to relapse, and people with mental illness often delay seeking care. Therefore, we are unlikely to capture the exact start date of the mental illnesses through registered diagnoses. Mental illness was identified through the presence of several ICD codes: non-affective psychotic disorders (ICD-9: 295, 297, 298, excluding 295H and 298B; ICD-10: F20-24, F28-29); affective psychotic disorders (ICD-9: 296, 295H, 298B; ICD-10: F25, F30-31, F32.3, F33.3); alcohol or drug misuse (ICD-9: 291, 292, 303, 304; ICD-10: F10-16, F18-19, excluding fourth digit 0 and 9); mood disorders, excluding those with psychotic symptoms (ICD-9: 300E, 311; ICD-10: F32-34, F38-39, excluding F32.3 and F33.3); anxiety and stress related disorders (ICD-9: 300A-D, 300F-H, 300W-X, 306, 307A, 308, 309; ICD-10: F40-48); eating disorders (ICD-9: 307B, 307F; ICD-10: F50); and personality disorders (ICD-9: 301; ICD-10: F60-63, F68-69).

In our study, we considered non-affective and affective psychotic disorders to be serious mental illness, while mood, anxiety, and stress related disorders were considered to be common mental disorders.

### Childhood injuries

We defined childhood injuries based on ICD codes for the external cause of injury and the intent, which were recorded during an inpatient or outpatient visit identified from the National Patient Register. Unintentional injuries included transport injuries (ICD-9: E800-849; ICD-10: V01-99); falls (ICD-9: E880-888; ICD-10: W00-19); burns (ICD-9: E890-899, E919, E924; ICD-10: X00-19); drowning and suffocation (ICD-9: E910-913; ICD-10: W65-84); and poisoning (ICD-9: E850-869; ICD-10: X40-49). Intentional injuries included violence related injuries, such as interpersonal assault (ICD-9: E960-966, 968-969; ICD-10: X85-Y05, Y08-09), and maltreatment syndromes, such as physical, psychological, and sexual abuse (ICD-9: E967; ICD-10: Y06-07). If the time between two visit dates of the same injury group was less than or equal to 30 days, we counted these as one occurrence, indexed by the earliest visit date.

### Other covariates

#### Demographic characteristics

We obtained demographic characteristics from the Total Population Register: the child’s sex, birth year, and number of siblings; country of birth of the child and the parents (categorised as Sweden or other countries); and parental age at the time of the child’s birth. The Longitudinal Integration Database for Health Insurance and Labour Market Studies provided information on children’s living arrangements at the beginning of each developmental period. We categorised living arrangements as living with both birth parents, living with one birth parent (which included children living with one birth parent and one step parent), or living with neither birth parent.

#### Parental socioeconomic position

We obtained the following indicators of parental socioeconomic position from the Longitudinal Integration Database for Health Insurance and Labour Market Studies: parental education, employment status, household receipt of social welfare benefits, and household disposable income. These data were extracted for the year before the start year of each developmental period. We defined parental education as the parent’s highest educational attainment, which was categorised into compulsory (0-9 years), secondary (10-12 years), and university (≥13 years). Parental employment status was categorised as a parent having gainful employment or not. We defined household receipt of social welfare benefits as at least one parent having received need based financial assistance from the municipality (yes or no). Household disposable income was defined as the annual sum of income and public benefits earned by all family members, adjusted for taxation. For analysis, we categorised this income into fifths for each year.

#### Childhood psychopathology

We defined childhood psychopathology for each developmental period as the presence of an inpatient or outpatient visit with a mental illness diagnosis, which was identified through the National Patient Register (ICD codes listed in supplementary table 6).

### Statistical analysis

We calculated absolute and relative risk measurements to compare the risk of injuries among children and adolescents with parental mental illness (CAPRI) and those without parental mental illness (non-CAPRI) within each developmental period. We estimated absolute measurements (rate differences) by using Poisson regression in additive scale.^40^ Relative measurements (rate ratios) were estimated by using Poisson regression. To account for clustering in siblings, these measurements were fitted using generalised estimating equations with an exchangeable correlation structure.

We controlled for the following variables: sex, birth year (continuous), number of siblings (continuous), parental country of birth, maternal age at birth (continuous), paternal age at birth (continuous), and living arrangements. Additionally, we controlled for parental education, parental employment status, household income fifth (categorical), and follow-up year. Missing observations in demographic and socioeconomic variables, ranging from 0.0% for parental country of birth to 19.6% for parental employment status in children aged 0-1, were included as a separate category in the analyses. However, we excluded 11 children (0.0%) who had missing data in all covariates. Analyses were conducted overall for any parental mental illness that has been recorded in the registers during a specific developmental age period, and by type of maternal and paternal mental illness during that period. If the parents had multiple mental disorder diagnoses, they were included in each diagnostic category (that is, they were considered to be exposed for different diagnoses). We also conducted analyses separately for each category of injury.

Children with mental health problems have been reported to have higher rates of unintentional injuries,^41^ and it has been suggested that childhood psychopathology might partly explain the association between parental mental illness and childhood injuries.^16^ ^23^ To assess this possibility, we conducted a supplementary analysis in which we repeated the main analyses with further adjustment for child psychopathology during specific child age periods.

We conducted two additional sensitivity analyses. Firstly, we examined the robustness of our results to the possibility of reverse causality by defining exposure to include the five years before each specific developmental period. Secondly, we restricted the analysis to children known to be living with their parents because they could represent the most conservatively defined exposure group (children directly affected by the parental mental condition). Therefore, this analysis might serve to evaluate the robustness of the association. Data management and analyses were performed using SAS version 9.4 and R version 3.6.1.

### Patient and public involvement

We did not include patient and public directly throughout the research process (formulation of research questions, outcome measures development, study design, recruitment, the conduct of the study, and dissemination of the results).

## Results

### Demographic and socioeconomic characteristics of the study population

Table 1 shows the distribution of demographic characteristics of the children and their parents by the presence of parental mental illness at any time during follow-up. A lower proportion of CAPRI (3.5%) than non-CAPRI (4.9%) were born outside Sweden, whereas a higher proportion of CAPRI (12.7%) than non-CAPRI (9.8%) had at least one parent born outside Sweden. On average, CAPRI were born to younger parents than non-CAPRI. The mean maternal age among CAPRI was 29.5 years (standard deviation 5.7) and the mean paternal age was 32.7 years (6.7); among non-CAPRI, the mean maternal and paternal age was 30.4 (5.1) and 33.3 (6.0) years, respectively.

**Table 1:** Selected characteristics of children in the study population (n=1 542 000), according to parental mental illness exposure. Data are number (%) of children

Variables and categories	Children aged 0-17 with any parental mental illness (n=1 542 000)	
	Yes (CAPRI; n=330 791)	No (non-CAPRI; n=1 211 209)
**Characteristics of children**		
Sex:		
Male	170 983 (51.7)	622 033 (51.4)
Female	159 808 (48.3)	589 176 (48.6)
Country of birth:		
Other countries	11 568 (3.5)	59 550 (4.9)
Sweden	319 223 (96.5)	1 151 659 (95.1)
Birth year:		
1996	20 015 (6.1)	75 049 (6.2)
1997	20 117 (6.1)	70 723 (5.8)
1998	20 390 (6.2)	69 341 (5.7)
1999	20 487 (6.2)	68 241 (5.6)
2000	20 779 (6.3)	69 521 (5.7)
2001	20 384 (6.2)	69 791 (5.8)
2002	20 962 (6.3)	72 684 (6.0)
2003	21 221 (6.4)	74 746 (6.2)
2004	20 965 (6.3)	76 407 (6.3)
2005	20 742 (6.3)	76 213 (6.3)
2006	21 514 (6.5)	78 919 (6.5)
2007	21 277 (6.4)	79 348 (6.6)
2008	21 227 (6.4)	80 316 (6.6)
2009	20 892 (6.3)	82 001 (6.8)
2010	20 876 (6.3)	85 160 (7.0)
2011	18 943 (5.7)	82 749 (6.8)
No of siblings (mean (SD))	1.6 (1.0)	1.5 (0.9)
**Family characteristics**		
Parental country of birth:		
All known parents born outside Sweden	59 620 (18.0)	219 224 (18.1)
All known parents born in Sweden	229 094 (69.3)	873 664 (72.1)
One parent born outside and one parent born inside Sweden	42 076 (12.7)	118 311 (9.8)
Missing	1 (0.0)	10 (0.0)
Maternal age at birth (mean (SD))	29.5 (5.7)	30.4 (5.1)
Paternal age at birth (mean (SD))	32.7 (6.7)	33.3 (6.0)

Supplementary table 1 shows the distribution of parental socioeconomic status of children in the study population from childhood to adolescence by the presence of parental mental illness during each developmental age period. Overall, CAPRI were more likely than non-CAPRI to live with only one parent or with neither parent, and to live in households in the lower income groups or receiving public assistance; their parents were more likely to be unemployed or less educated.

Mental illness was more common among mothers than fathers in all age groups of children (supplementary table 2). Anxiety and stress related disorders were the most common diagnostic groups among both parents, followed by other mood disorders.

### Parental mental illness and risk of injuries among the offspring

Table 2 shows rates of injuries and rate differences between CAPRI and non-CAPRI during each developmental age period. Overall, injuries were more common among CAPRI than non-CAPRI in all age groups, and the difference was higher during the first year of life (CAPRI had 2088 additional injuries per 100 000 person years compared with non-CAPRI at age 0-1 year). Falls were the most common type of injury in all age groups, peaking at age 10-12 years (CAPRI 5443/100 000 person years, non-CAPRI 4948/100 000 person years). Burns were the second most common type of injury followed by poisoning; both peaked at age 0-1 year (burns rate for CAPRI 479/100 000 person years, non-CAPRI 285/100 000 person years; poisoning rate for CAPRI 228/100 000 person years, non-CAPRI 119/100 000 person years) and decreased with child age. By contrast, the rate of transport injuries increased with child age, with the highest rate occurring in adolescence (CAPRI 1494/100 000 person years, non-CAPRI 1383/100 000 person years). The rate of violence related injury among CAPRI and non-CAPRI peaked in adolescence (CAPRI 364/100 000 person years, non-CAPRI 152/100 000 person years). However, CAPRI also had another peak in violence related injury during the first year of life (44/100 000 person years). Overall, the most common causes of violence related injuries were assault by unarmed fight, maltreatment syndromes (including physical, sexual, and psychological abuse), and sexual assault (data not shown).

**Table 2:** Number of injuries (rate of injuries) and rate differences of childhood injuries by age of the child and the presence of parental mental illness (n=1 542 000)

	Period 1: 0-1 year (n=1 351 683)				Period 2: 2-5 years (n=1 458 868)				Period 3: 6-9 years (n=1 371 158)				Period 4: 10-12 years (n=973 174)				Period 5: 13-17 years (n=700 454)		
	CAPRI (n=10 345)	Non-CAPRI (n=1 248 238)	Rate difference (95% CI)		CAPRI (n=181 043)	Non-CAPRI (n=1 277 825)	Rate difference (95% CI)		CAPRI (n=206 425)	Non-CAPRI (n=1 164 733)	Rate difference (95% CI)		CAPRI (n=153 617)	Non-CAPRI (n=819 557)	Rate difference (95% CI)		CAPRI (n=124 655)	Non-CAPRI (n=575 799)	Rate difference (95% CI)
Person years	201 670.5	2 434 161.5	—		682 139.3	4 848 731.0	—		688 808.1	3 887 451.8	—		389 982.3	2 060 588.3	—		434 506.9	1 899 956.2	—
Any injury	10 235 (5075.1)	72 723 (2987.6)	2087.5 (1987.4 to 2188.8)		29 742 (4360.1)	163 793 (3378.1)	982.0 (930.0 to 1034.4)		31 881 (4628.4)	156 319 (4021.1)	607.3 (552.9 to 662.0)		25 893 (6639.5)	122 656 (5952.5)	687.1 (599.9 to 774.8)		27 888 (6418.3)	113 928 (5996.4)	422.0 (339.2 to 505.2)
Transport injury	157 (77.9)	1460 (60.0)	17.9 (5.9 to 31.0)		2135 (313.0)	11 638 (240.0)	73.0 (59.2 to 87.1)		4420 (641.7)	19 803 (509.4)	132.3 (112.2 to 152.6)		4314 (1106.2)	20 423 (991.1)	115.1 (79.7 to 151.1)		6493 (1494.3)	26 268 (1382.6)	111.8 (72.0 to 152.0)
Fall	8447 (4188.5)	60 195 (2472.9)	1715.6 (1624.7 to 1807.7)		25 083 (3677.1)	142 382 (2936.5)	740.6 (692.8 to 788.8)		26 866 (3900.4)	135 016 (3473.1)	427.2 (377.2 to 477.6)		21 225 (5442.6)	101 949 (4947.6)	495.0 (416.0 to 574.5)		19 617 (4514.8)	84 737 (4460.0)	54.8 (−14.9 to 125.0)
Burn	966 (479.0)	6944 (285.3)	193.7 (163.4 to 225.3)		1184 (173.6)	5156 (106.3)	67.2 (57.1 to 77.7)		426 (61.9)	1628 (41.9)	20.0 (13.9 to 26.3)		226 (58.0)	851 (41.3)	16.7 (8.9 to 25.0)		280 (64.4)	788 (41.5)	23.0 (15.1 to 31.3)
Drowning	166 (82.3)	1278 (52.5)	29.8 (17.6 to 43.3)		339 (49.7)	1603 (33.1)	16.6 (11.3 to 22.3)		116 (16.8)	427 (11.0)	5.9 (2.8 to 9.3)		60 (15.4)	189 (9.2)	6.2 (2.4 to 10.6)		68 (15.7)	169 (8.9)	6.8 (3.0 to 11.0)
Poisoning	459 (227.6)	2902 (119.2)	108.4 (87.7 to 130.3)		985 (144.4)	3688 (76.1)	68.3 (59.2 to 77.9)		141 (20.5)	541 (13.9)	6.6 (3.1 to 10.3)		74 (19.0)	247 (12.0)	7.0 (2.7 to 11.9)		269 (61.9)	637 (33.5)	28.4 (20.8 to 36.5)
Violence	88 (43.6)	167 (6.9)	36.8 (28.2 to 46.6)		224 (32.8)	416 (8.6)	24.3 (20.1 to 28.8)		251 (36.4)	567 (14.6)	21.9 (17.4 to 26.7)		355 (91.0)	762 (37.0)	54.1 (44.5 to 64.2)		1581 (363.9)	2888 (152.0)	211.9 (193.3 to 230.9)

CAPRI=children and adolescents with parental mental illness.

Number of injuries=summary number of injuries; rate of injuries=number of injuries per 100 000 person years; rate difference=injury rates in exposed children − injury rates in unexposed children (per 100 000 person years).

Supplementary table 3 shows rates of injuries and rate differences between CAPRI and non-CAPRI by type of parental mental illness. Overall, excess injury rates were higher for common mental disorders than for more serious mental illness. For example, children of parents with mood disorders had 2494 additional injuries per 100 000 person years at age 0-1 years, while children of parents with non-affective psychotic disorders had an additional 626 injuries per 100 000 person years for the same age group.

In crude and adjusted analyses, CAPRI had a higher risk of injuries than non-CAPRI at all ages (table 3). Crude rate ratios for the association between any parental mental illness and any childhood injury ranged from 1.70 (95% confidence interval 1.66 to 1.73) during the first year of life to 1.07 (1.05 to 1.09) during adolescence. Adjustment for confounders (table 3 and supplementary fig 2) attenuated the estimates, but most remained raised (adjusted rate ratio at 0-1 year 1.30, 95% confidence interval 1.26 to 1.33). The increased risk of injuries was higher for children exposed to parental common mental disorders (eg, adjusted rate ratio for mood disorders at 0-1 year 1.35, 95% confidence interval 1.29 to 1.41) than for those exposed to more serious mental illness (eg, adjusted rate ratio for non-affective psychotic disorders at 0-1 year 1.06, 95% confidence interval 0.91 to 1.24). For younger children (0-5 years), all types of parental mental illness except for non-affective psychotic disorders were associated with a higher risk of injuries. However, among older children, a higher risk of injuries was observed mostly for common mental disorders in parents. Overall, the effect of mental illness was greater for maternal than paternal exposure (eg, any maternal mental illness at 0-1 year, adjusted rate ratio 1.31, 95% confidence interval 1.26 to 1.35; any paternal mental illness at 0-1 year 1.27, 1.21 to 1.33). We did not conduct analyses for paternal eating disorders because few observations were made.

**Table 3:** Different types of parental mental illness and child’s risk of any injury (n=1 542 000). Values are rate ratios (95% confidence intervals)

	Period 1: 0-1 years			Period 2: 2-5 years			Period 3: 6-9 years			Period 4: 10-12 years			Period 5: 13-17 years	
	Crude	Adjusted*		Crude	Adjusted*		Crude	Adjusted*		Crude	Adjusted*		Crude	Adjusted*
**Parental mental illness†**														
Any mental illness	1.70 (1.66 to 1.73)	1.30 (1.26 to 1.33)		1.29 (1.27 to 1.31)	1.16 (1.14 to 1.18)		1.15 (1.13 to 1.17)	1.11 (1.09 to 1.13)		1.11 (1.10 to 1.13)	1.10 (1.08 to 1.12)		1.07 (1.05 to 1.09)	1.06 (1.04 to 1.08)
Non-affective psychotic disorders	1.20 (1.07 to 1.34)	1.06 (0.91 to 1.24)		1.02 (0.95 to 1.09)	0.98 (0.90 to 1.08)		1.01 (0.94 to 1.08)	1.02 (0.94 to 1.11)		0.93 (0.86 to 1.00)	0.94 (0.86 to 1.04)		0.87 (0.80 to 0.94)	0.91 (0.84 to 1.00)
Affective psychotic disorders	1.51 (1.39 to 1.64)	1.19 (1.06 to 1.34)		1.26 (1.21 to 1.32)	1.13 (1.07 to 1.19)		1.13 (1.08 to 1.17)	1.04 (0.99 to 1.09)		1.10 (1.05 to 1.15)	1.05 (1.00 to 1.11)		0.97 (0.92 to 1.01)	0.96 (0.91 to 1.01)
Alcohol or drug misuse	1.52 (1.44 to 1.61)	1.21 (1.12 to 1.31)		1.21 (1.17 to 1.25)	1.10 (1.05 to 1.15)		1.13 (1.10 to 1.17)	1.03 (0.98 to 1.07)		1.10 (1.06 to 1.14)	1.03 (0.99 to 1.08)		1.10 (1.06 to 1.14)	1.03 (0.98 to 1.07)
Mood disorders	1.81 (1.75 to 1.87)	1.35 (1.29 to 1.41)		1.29 (1.27 to 1.32)	1.15 (1.12 to 1.18)		1.15 (1.13 to 1.18)	1.11 (1.08 to 1.14)		1.13 (1.10 to 1.15)	1.11 (1.08 to 1.14)		1.05 (1.03 to 1.08)	1.04 (1.01 to 1.07)
Anxiety or stress related	1.77 (1.73 to 1.82)	1.31 (1.26 to 1.35)		1.33 (1.31 to 1.35)	1.18 (1.15 to 1.20)		1.17 (1.15 to 1.18)	1.12 (1.10 to 1.14)		1.14 (1.11 to 1.16)	1.13 (1.10 to 1.15)		1.10 (1.08 to 1.12)	1.09 (1.06 to 1.11)
Eating disorders	1.83 (1.67 to 2.01)	1.34 (1.18 to 1.52)		1.36 (1.26 to 1.47)	1.23 (1.11 to 1.35)		1.22 (1.12 to 1.31)	1.12 (1.02 to 1.23)		1.19 (1.09 to 1.31)	1.03 (0.92 to 1.15)		1.16 (1.04 to 1.29)	1.04 (0.91 to 1.18)
Personality disorders	1.72 (1.61 to 1.84)	1.35 (1.22 to 1.49)		1.36 (1.30 to 1.42)	1.24 (1.17 to 1.32)		1.20 (1.15 to 1.25)	1.08 (1.02 to 1.15)		1.14 (1.09 to 1.20)	1.06 (1.00 to 1.13)		1.10 (1.04 to 1.15)	1.00 (0.94 to 1.07)
**Maternal mental illness‡**														
Any mental illness	1.74 (1.70 to 1.79)	1.31 (1.26 to 1.35)		1.31 (1.29 to 1.33)	1.18 (1.15 to 1.20)		1.17 (1.15 to 1.18)	1.12 (1.09 to 1.14)		1.11 (1.08 to 1.13)	1.08 (1.06 to 1.11)		1.08 (1.06 to 1.10)	1.06 (1.03 to 1.09)
Non-affective psychotic disorders	1.13 (0.96 to 1.32)	0.87 (0.69 to 1.11)		1.02 (0.93 to 1.13)	1.03 (0.91 to 1.17)		1.01 (0.92 to 1.10)	1.04 (0.92 to 1.17)		0.88 (0.79 to 0.98)	0.89 (0.78 to 1.02)		0.86 (0.77 to 0.96)	0.94 (0.83 to 1.07)
Affective psychotic disorders	1.60 (1.45 to 1.77)	1.18 (1.02 to 1.37)		1.27 (1.21 to 1.35)	1.13 (1.05 to 1.21)		1.17 (1.11 to 1.23)	1.09 (1.02 to 1.16)		1.08 (1.03 to 1.14)	1.03 (0.96 to 1.10)		0.95 (0.90 to 1.01)	0.95 (0.88 to 1.01)
Alcohol or drug misuse	1.69 (1.55 to 1.85)	1.31 (1.13 to 1.52)		1.20 (1.13 to 1.27)	1.05 (0.96 to 1.14)		1.10 (1.04 to 1.17)	0.95 (0.88 to 1.03)		1.03 (0.97 to 1.10)	0.99 (0.91 to 1.07)		1.09 (1.02 to 1.15)	0.99 (0.92 to 1.07)
Mood disorders	1.83 (1.77 to 1.90)	1.35 (1.28 to 1.41)		1.31 (1.28 to 1.34)	1.16 (1.12 to 1.19)		1.17 (1.14 to 1.19)	1.11 (1.08 to 1.15)		1.11 (1.08 to 1.14)	1.08 (1.05 to 1.12)		1.05 (1.03 to 1.08)	1.03 (1.00 to 1.07)
Anxiety or stress related	1.81 (1.75 to 1.86)	1.32 (1.27 to 1.38)		1.34 (1.31 to 1.36)	1.18 (1.16 to 1.21)		1.17 (1.15 to 1.20)	1.12 (1.10 to 1.15)		1.13 (1.10 to 1.15)	1.10 (1.07 to 1.13)		1.10 (1.08 to 1.13)	1.08 (1.05 to 1.11)
Eating disorders	1.82 (1.65 to 2.00)	1.31 (1.16 to 1.49)		1.36 (1.26 to 1.47)	1.24 (1.12 to 1.37)		1.22 (1.13 to 1.32)	1.13 (1.03 to 1.25)		1.19 (1.08 to 1.30)	1.02 (0.91 to 1.14)		1.14 (1.03 to 1.27)	1.01 (0.89 to 1.16)
Personality disorders	1.77 (1.63 to 1.93)	1.35 (1.20 to 1.52)		1.39 (1.32 to 1.46)	1.27 (1.17 to 1.37)		1.20 (1.14 to 1.27)	1.06 (0.98 to 1.15)		1.13 (1.06 to 1.20)	1.04 (0.97 to 1.13)		1.09 (1.02 to 1.16)	1.00 (0.92 to 1.08)
**Paternal mental illness§**														
Any mental illness	1.61 (1.56 to 1.67)	1.27 (1.21 to 1.33)		1.25 (1.22 to 1.27)	1.14 (1.11 to 1.17)		1.12 (1.10 to 1.15)	1.10 (1.07 to 1.12)		1.11 (1.09 to 1.14)	1.11 (1.08 to 1.14)		1.05 (1.03 to 1.08)	1.06 (1.03 to 1.09)
Non-affective psychotic disorders	1.29 (1.09 to 1.51)	1.24 (1.01 to 1.53)		1.02 (0.93 to 1.12)	0.94 (0.83 to 1.07)		1.02 (0.92 to 1.12)	1.00 (0.89 to 1.12)		0.98 (0.88 to 1.09)	1.00 (0.88 to 1.13)		0.87 (0.79 to 0.97)	0.89 (0.79 to 1.01)
Affective psychotic disorders	1.39 (1.22 to 1.59)	1.20 (1.01 to 1.43)		1.25 (1.16 to 1.34)	1.13 (1.03 to 1.24)		1.04 (0.97 to 1.12)	0.96 (0.88 to 1.04)		1.12 (1.04 to 1.20)	1.08 (0.99 to 1.17)		0.98 (0.91 to 1.06)	0.98 (0.90 to 1.06)
Alcohol or drug misuse	1.46 (1.38 to 1.56)	1.20 (1.09 to 1.31)		1.22 (1.17 to 1.26)	1.11 (1.05 to 1.17)		1.15 (1.10 to 1.19)	1.06 (1.01 to 1.12)		1.12 (1.07 to 1.16)	1.06 (1.00 to 1.11)		1.09 (1.04 to 1.13)	1.04 (0.98 to 1.09)
Mood disorders	1.74 (1.65 to 1.84)	1.33 (1.24 to 1.42)		1.25 (1.22 to 1.29)	1.14 (1.09 to 1.18)		1.13 (1.10 to 1.17)	1.11 (1.07 to 1.15)		1.14 (1.10 to 1.18)	1.14 (1.09 to 1.18)		1.04 (1.00 to 1.08)	1.06 (1.01 to 1.10)
Anxiety or stress related	1.69 (1.62 to 1.77)	1.25 (1.18 to 1.33)		1.30 (1.27 to 1.33)	1.16 (1.12 to 1.20)		1.14 (1.11 to 1.17)	1.11 (1.08 to 1.14)		1.14 (1.11 to 1.18)	1.16 (1.12 to 1.20)		1.08 (1.05 to 1.11)	1.09 (1.06 to 1.13)
Personality disorders	1.59 (1.42 to 1.78)	1.31 (1.12 to 1.54)		1.30 (1.21 to 1.39)	1.20 (1.09 to 1.31)		1.21 (1.13 to 1.29)	1.11 (1.02 to 1.21)		1.14 (1.06 to 1.23)	1.09 (0.99 to 1.19)		1.10 (1.02 to 1.19)	1.01 (0.92 to 1.12)

* Adjusted for sex, birth year, number of siblings (square terms), parental country of birth (missing excluded), maternal age at birth (square terms), paternal age at birth (square terms), living arrangements, parental education, parental employment status, household income.

† Period 1, n=1 351 683; period 2, n=1 458 868; period 3, n=1 371 158; period 4, n=973 174; period 5, n=700 454.

‡ Period 1, n=1 350 054; period 2, n=1 456 895; period 3, n=1 368 854; period 4, n=970 751; period 5, n=697 550.

§ Period 1, n=1 333 264; period 2, n=1 440 026; period 3, n=1 353 299; period 4, n=956 468; period 5, n=682 002.

Table 4 and supplementary figure 3 show the associations between any parental mental illness and different categories of childhood injuries. CAPRI had higher adjusted rate ratios for almost all types of injuries, especially at 0-5 years. During the first years of life, adjusted rate ratios were highest for violence related injuries (0-1 year: 3.54, 95% confidence interval 2.28 to 5.48; 2-5 years: 3.02, 2.20 to 4.13), followed by poisoning (0-1 year: 1.79, 1.56 to 2.04; 2-5 years: 1.70, 1.55 to 1.87). The risk of fall related injury was highest during the first year of life (0-1 year: 1.28, 1.24 to 1.32) and reduced thereafter. Preschool and adolescent CAPRI were at highest risk of burn injuries (2-5 years: 1.39, 1.26 to 1.53; 13-17 years: 1.47, 1.22 to 1.76). The risk of drowning and suffocation among CAPRI increased with age (13-17 years: 1.79, 1.08 to 2.96). Finally, the risk of transport injuries was only slightly higher among CAPRI than non-CAPRI at all ages.

**Table 4:** Parental mental illness and child’s risk of various injuries (n=1 542 000). Values are rate ratios (95% confidence intervals)

Type of injury	Period 1: 0-1 years			Period 2: 2-5 years			Period 3: 6-9 years			Period 4: 10-12 years			Period 5: 13-17 years	
	Crude	Adjusted*		Crude	Adjusted*		Crude	Adjusted*		Crude	Adjusted*		Crude	Adjusted*
**Any parental mental illness†**														
Transport injury	1.30 (1.10 to 1.53)	1.09 (0.86 to 1.38)		1.30 (1.24 to 1.37)	1.09 (1.02 to 1.16)		1.26 (1.22 to 1.31)	1.18 (1.13 to 1.23)		1.12 (1.07 to 1.16)	1.09 (1.04 to 1.14)		1.08 (1.04 to 1.12)	1.06 (1.02 to 1.11)
Fall	1.69 (1.65 to 1.73)	1.28 (1.24 to 1.32)		1.25 (1.23 to 1.27)	1.14 (1.12 to 1.16)		1.12 (1.11 to 1.14)	1.09 (1.07 to 1.11)		1.10 (1.08 to 1.12)	1.09 (1.07 to 1.11)		1.01 (0.99 to 1.03)	1.03 (1.00 to 1.05)
Burn	1.68 (1.56 to 1.81)	1.30 (1.18 to 1.43)		1.63 (1.52 to 1.76)	1.39 (1.26 to 1.53)		1.48 (1.31 to 1.67)	—¶		1.40 (1.18 to 1.67)	1.14 (0.93 to 1.41)		1.55 (1.33 to 1.81)	1.47 (1.22 to 1.76)
Drowning	1.57 (1.32 to 1.86)	1.24 (0.99 to 1.54)		1.50 (1.33 to 1.70)	1.32 (1.14 to 1.54)		1.53 (1.22 to 1.92)	1.27 (0.94 to 1.72)		1.68 (1.25 to 2.25)	—¶		1.76 (1.23 to 2.53)	1.79 (1.08 to 2.96)
Poisoning	1.91 (1.73 to 2.11)	1.79 (1.56 to 2.04)		1.89 (1.75 to 2.03)	1.70 (1.55 to 1.87)		1.47 (1.19 to 1.81)	—¶		1.58 (1.19 to 2.09)	1.48 (1.06 to 2.05)		1.84 (1.58 to 2.15)	1.45 (1.21 to 1.73)
Violence	6.23 (4.52 to 8.59)	3.54 (2.28 to 5.48)		3.77 (3.08 to 4.63)	3.02 (2.20 to 4.13)		2.47 (2.07 to 2.94)	1.51 (1.21 to 1.89)		2.45 (2.14 to 2.80)	1.55 (1.30 to 1.84)		2.39 (2.23 to 2.56)	1.60 (1.47 to 1.74)
**Any maternal mental illness‡**														
Transport injury	1.36 (1.12 to 1.66)	1.05 (0.78 to 1.40)		1.27 (1.20 to 1.35)	1.07 (1.00 to 1.16)		1.26 (1.20 to 1.31)	1.16 (1.10 to 1.22)		1.13 (1.08 to 1.18)	1.10 (1.05 to 1.16)		1.08 (1.03 to 1.12)	1.07 (1.02 to 1.12)
Fall	1.73 (1.68 to 1.78)	1.28 (1.23 to 1.32)		1.27 (1.25 to 1.29)	1.15 (1.12 to 1.17)		1.14 (1.12 to 1.16)	1.10 (1.08 to 1.13)		1.08 (1.06 to 1.11)	1.07 (1.04 to 1.10)		1.02 (1.00 to 1.05)	1.03 (1.00 to 1.06)
Burn	1.74 (1.59 to 1.89)	1.36 (1.21 to 1.52)		1.69 (1.55 to 1.84)	1.45 (1.29 to 1.62)		1.63 (1.42 to 1.86)	1.41 (1.20 to 1.65)		1.50 (1.21 to 1.84)	1.23 (0.96 to 1.57)		1.52 (1.28 to 1.82)	1.44 (1.17 to 1.78)
Drowning	1.59 (1.30 to 1.95)	1.43 (1.11 to 1.84)		1.59 (1.39 to 1.83)	1.40 (1.18 to 1.67)		1.57 (1.23 to 2.00)	1.32 (0.97 to 1.78)		1.67 (1.18 to 2.36)	—¶		1.13 (0.76 to 1.67)	0.95 (0.59 to 1.52)
Poisoning	2.08 (1.85 to 2.33)	1.92 (1.65 to 2.24)		1.97 (1.81 to 2.14)	1.85 (1.67 to 2.05)		1.47 (1.16 to 1.87)	—¶		1.67 (1.22 to 2.30)	1.60 (1.10 to 2.32)		1.78 (1.49 to 2.12)	1.34 (1.08 to 1.66)
Violence	5.76 (4.06 to 8.19)	3.43 (2.07 to 5.69)		4.09 (3.23 to 5.17)	3.69 (2.60 to 5.24)		2.83 (2.32 to 3.46)	1.84 (1.43 to 2.37)		2.44 (2.10 to 2.84)	1.51 (1.24 to 1.84)		2.38 (2.20 to 2.56)	1.61 (1.46 to 1.78)
**Paternal mental illness§**														
Transport injury	1.15 (0.88 to 1.50)	1.05 (0.74 to 1.50)		1.36 (1.27 to 1.46)	1.13 (1.03 to 1.23)		1.25 (1.19 to 1.32)	1.19 (1.12 to 1.27)		1.10 (1.04 to 1.16)	1.09 (1.02 to 1.15)		1.07 (1.02 to 1.12)	1.07 (1.01 to 1.13)
Fall	1.61 (1.55 to 1.67)	1.27 (1.21 to 1.34)		1.21 (1.18 to 1.24)	1.12 (1.09 to 1.16)		1.09 (1.07 to 1.12)	1.08 (1.05 to 1.10)		1.10 (1.08 to 1.13)	1.11 (1.08 to 1.15)		0.99 (0.96 to 1.02)	1.03 (0.99 to 1.06)
Burn	1.56 (1.40 to 1.75)	1.19 (1.03 to 1.38)		1.52 (1.37 to 1.68)	1.26 (1.10 to 1.44)		1.35 (1.12 to 1.62)	—¶		1.31 (1.04 to 1.65)	—¶		1.45 (1.18 to 1.79)	1.31 (1.03 to 1.67)
Drowning	1.59 (1.25 to 2.03)	1.05 (0.74 to 1.49)		1.28 (1.07 to 1.54)	1.09 (0.87 to 1.37)		1.35 (0.94 to 1.94)	0.98 (0.57 to 1.68)		1.53 (1.02 to 2.30)	—¶		2.25 (1.36 to 3.74)	2.41 (1.20 to 4.83)
Poisoning	1.81 (1.56 to 2.11)	1.61 (1.32 to 1.96)		1.79 (1.61 to 1.99)	1.47 (1.28 to 1.69)		1.49 (1.14 to 1.95)	—¶		1.56 (1.07 to 2.28)	1.44 (0.94 to 2.21)		1.94 (1.61 to 2.35)	1.51 (1.20 to 1.90)
Violence	6.66 (4.32 to 10.25)	2.81 (1.54 to 5.11)		3.21 (2.48 to 4.16)	1.64 (1.09 to 2.45)		2.46 (1.92 to 3.14)	1.40 (1.03 to 1.89)		2.33 (1.95 to 2.79)	1.43 (1.14 to 1.79)		2.32 (2.12 to 2.53)	1.48 (1.33 to 1.65)

* Adjusted for sex, birth year, number of siblings (square terms), parental country of birth (missing excluded), maternal age at birth (square terms), paternal age at birth (square terms), living arrangements, parental education, parental employment status, household income.

† Period 1, n=1 351 683; period 2, n=1 458 868; period 3, n=1 371 158; period 4, n=973 174; period 5, n=700 454.

‡ Period 1, n=1 350 054; period 2, n=1 456 895; period 3, n=1 368 854; period 4, n=970 751; period 5, n=697 550.

§ Period 1, n=1 333 264; period 2, n=1 440 026; period 3, n=1 353 299; period 4, n=956 468; period 5, n=682 002.

¶ Estimates could not be obtained because of the low number of observations.

### Sensitivity and supplementary analyses

When we restricted exposure to parental illness recorded before the outcome period and excluded those exposed within the outcome period, little material effect was noted on the associations between parental mental illness and offspring’s risk of injuries (eg, adjusted rate ratio at 0-1 year 1.28, 95% confidence interval 1.24 to 1.33; supplementary table 4). Additionally, the results did not materially change when we restricted the analysis to children who lived with their parent(s) (supplementary table 5). Finally, we found a higher proportion of CAPRI had a diagnosis of child psychopathology than non-CAPRI (13.9% *v* 6.2% for all ages), however adjustment for child psychopathology did not affect the results (data not shown).

## Discussion

### Key results

This large study examines preventable injuries in children exposed to parental mental illness. We report an increase in the risk of injuries in CAPRI starting from birth and extending throughout childhood into adolescence. Taking account of detailed demographic and socioeconomic factors had little influence on this finding.

Exposure to parental mental illness in the first year of life provided the greatest excess risk: approximately 30% increase for any type of injury or a rate of more than 2000 injuries per 100 000 person years. Because of the large population rates of injuries, these findings are of clinical and public health significance. The risk increase was especially large for violence related injuries, poisoning, and burns compared with lower estimates for falls, drowning and suffocation, and transport injuries. We found that exposure to maternal mental illness resulted in a slightly higher risk for children overall than exposure to paternal mental illness. We also found that exposure to common mental disorders such as parental depression and anxiety had a greater influence on the risk of injuries than exposure to serious parental mental illness, such as schizophrenia.

### Comparison with previous studies

Our findings are comparable to several recent UK studies. The largest previous study examining associations between mental illness in the household and childhood risk of injuries used electronic health records of 253 717 children in Wales.^11^ The study reported a 14% increase in the risk of emergency hospital admissions because of injuries and a 55% increase in the risk of admissions because of victimisation among children living with adults with mental illness.^11^ Another UK study reported that maternal depression and self-reported psychological distress were associated with increased risk of injuries in offspring aged 3-11 years,^14^ with the increase greatest in children aged 3-5 years. Another reported an increased risk of burns and poisoning in children aged 0-4 years associated with maternal depression.^16^ Our findings also correspond with a Finnish study that reported parental substance misuse increased the overall risk of hospitalisation from injuries in children aged 0-6 years.^15^ Additionally, our findings are in line with a UK study that found a higher risk of injuries among children of mothers with perinatal depression.^19^

Our study makes an important contribution by addressing key limitations in the previous literature and showing that maternal and paternal exposure increases the risk of preventable child injury. Additionally, we were able to show the risk for different types of mental illness and injury, and to show the risk for different age groups. Notably, our estimates of risk are generally higher than in the Welsh population study,^11^ probably because of differences in determining exposure and outcome, and because of variation in data sources.

### Possible explanations of our findings

The mechanism explaining how parental mental illness increases the risk of injuries among children is likely to be complex. Existing literature speculates that parents with a mental illness are less able to be responsive to the child and its environment.^42^ ^43^ Alongside such notions is the understanding that some parents with mental illness could find it harder to be vigilant and to maintain parental supervision. This might be especially important during the first years of life when the child is beginning to crawl and walk, and needs a great deal of attention.^42^ ^43^ However, a lessening of parental supervision as an explanation of heightened child injury risk might not only apply to parents with mental illness but could also occur in families with other types of parental ill health. For example, a Swedish study found an increased risk of injuries among children of parents who had been newly diagnosed with cancer.^44^

Alternatively, it is possible that parents with mental illness are less likely or able to take up and implement simple safety practices. Previous studies suggest that maternal depression might be associated with a lack of uptake of parental prevention practices,^45^ which extends from mothers who are ill being much less likely to take up sudden infant death syndrome prevention messages, such as smoking cessation during pregnancy.^46^

Active maltreatment in CAPRI^15^ ^47^ might partly explain their markedly higher risk of injury from violence throughout their childhood. This is in agreement with the patterns of excess risks of harm to infants whose mothers have been admitted to mother and baby units and population studies of premature mortality.^48^ ^49^ Both studies imply that risks are greater for offspring of parents with depression or substance misuse,^48^ ^49^ as opposed to more serious mental illnesses such as schizophrenia for which risks were generally lower. The violence related injury was a rare outcome in our study compared with other types of injuries in spite of the increased risk of violence for CAPRI, particularly for infants and adolescents. Further, we did not have information about whether parents themselves inflicted the violence on their children; and we did not identify whether children experienced violence at home or elsewhere.

Certain psychotropic medications with sedative effects, such as benzodiazepines, have been associated with a higher risk of injuries, including road traffic crashes^50^ and falls.^51^ Therefore, it is hypothetically plausible that some of the excess risks of child injury observed in our study might be related to psychotropic medication use among parents, which has not been previously studied.

Deprivation and lack of social capital are known to be associated with mental illness^7^ and child injuries, including violence.^6^ ^52^ ^53^ Therefore, children who have parents with mental illness might have increased risk of injuries as an indirect result of socioeconomic influences.^22^ ^23^ Adjustment for a range of individual level socioeconomic factors including parental education, employment, and income had little influence on the risks found in our study. However, these socioeconomic indicators might not capture all of the important social and contextual factors that contribute to the risk of injury in children of parents with mental illness. For example, families with parental mental illness might be more likely to live in less affluent, more violent, and potentially more dangerous areas, which might contribute to the additional risk of injuries seen in these children.

Finally, because our estimates were very similar after we adjusted for childhood psychopathology, our study does not support the notion that behavioural problems in a child mediate the effect of parental mental illness on the risk of injuries.^16^ ^23^ Although rates of injuries were higher with increasing age of the child, the association with parental mental illness was strongest during the first years of life and weak during adolescence when offspring are more independent of their parents. Additionally, other factors such as peer relations play a bigger role in safety.^21^ Our findings indicate that parental factors, including an ability to put preventive measures into place and supervising children sufficiently during the first years of life, might explain the excess risk of injuries in CAPRI, rather than the children’s behaviour.

### Strengths and limitations

This study has several strengths, including the large, population cohort and data from high quality national registers with few missing data. The large power generated from this sample allowed us to present associations for a wide range of exposures and outcomes. Additionally, exposure and outcome were determined using clinical diagnosis, unlike most previous studies.^13^ ^14^ ^17^ ^18^ ^20^ ^21^ ^22^ ^23^ We obtained exposure information for the fathers, which was not available in most previous papers. Finally, we were able to adjust for many confounders, including socioeconomic indicators at different ages of the child.

However, several limitations remain. Firstly, our exposure only included clinical diagnoses made within secondary care; we did not have information on people treated exclusively within primary care, or those who did not seek treatment. Therefore, we captured more severe conditions but might have missed a large proportion of less severe common mental disorders in parents because these are often treated in primary care. However, a recent study in Sweden showed that 20% of patients with common mental disorders treated in primary care are jointly treated in secondary care,^54^ and so we might have captured a fraction of these patients. Further, only a small number of people seek help for parental addiction.^55^ Additionally, we do not know if parental mental illness influenced treatment seeking among children or the likelihood of them presenting to services (getting into the data) with an injury. Relying on register data alone means we were unable to examine factors such as quality of parenting and parental supervision, which could explain some of the reported associations. Further, we did not have detailed information on the circumstances surrounding the injury outcome, such as the perpetrators when violence related injury occurred (that is, whether it was a family member or others). Finally, we probably did not capture all the social and contextual factors that could have influenced the association between parental mental illness and risk of injuries in offspring, such as housing conditions and neighbourhood characteristics.^56^

### Implications

Our study is important because it highlights very specific risks for CAPRI across childhood into adolescence. Our findings suggest that increasing access to parental support for parents with mental illness might be beneficial for child injury prevention, especially in the first few years after the child is born. Our study also shows the importance of supporting mothers and fathers as the care givers of the children. Parental support could include facilitating access to practical information and strengthening family routines and actions about child safety. Existing evidence shows that targeted home safety interventions, including parental support, might improve family prevention practices and reduce injury rates among children.^57^ ^58^ This kind of intervention could be tailored to suit the needs of parents who experience mental illness. In Sweden, the *National Handbook for Child Health Services Professionals* includes a guideline on providing information about child safety to all parents through home visits and visits to the child health care centre, and extra psychological support to families who might need it.^59^ However, the extent to which these guidelines are implemented in practice is currently unknown and probably varies from region to region. Most importantly, future studies need to evaluate the implementation of such targeted home safety interventions in families with parental mental illness and identify the specific needs and experiences of these families.^60^ ^61^

Our findings also suggest that early screening and treatment of perinatal mental illness in mothers and fathers might reduce the child’s risk of injury, including the risk of exposure to violence. However, it should be noted that most people with mental illness do not have violent behaviour.^62^ Nevertheless, the evidence shows that effective management and treatment of mental illness, for example with certain types of psychotropic medication such as antipsychotics and mood stabilisers, reduces the rates of violence among people with mental illness.^63^ Practical provision of mental healthcare needs to take into account the increased risk of injuries because of violence in children of parents with mental illness and parenting interventions aimed at reducing the burden of parental mental illness should also address injury risk.

Our study revealed that the risk of injuries among children exposed to parental mental illness was more pronounced for common parental mental illness than for serious mental illness, and this was particularly true among older children. This finding highlights the importance of recognising and treating common mental illnesses among parents during all child developmental periods. Our findings might also help to reevaluate a presumed inadequacy of the parenting capacity of those with serious mental illness, which was suggested by previous studies.^48^ Our study also shows a need to evenly distribute resources for parents with different types of mental illness in the context of child injury prevention.

### Conclusions

This study shows that parental mental illness, which is common in families in the general population, is associated with an increased risk of injuries among offspring from birth through adolescence. The risk is particularly high during the first year of a child’s life. The increased risk is evident for maternal and paternal exposure, and for all types of parental mental illness. These findings suggest there could be potential benefits to child injury prevention of increasing access to parental support for parents who are mentally ill, and recognising and treating perinatal mental illness among parents in secondary care.

**What is already known on this topic**

Previous studies have shown associations between parental mental illness and the risk of injuries in offspring
Most studies have focused on maternal exposure, common mental disorders, and younger children, or were unable to separate risks by type of injuries

**What this study adds**

Exposure to parental mental illness is associated with increased risk of injuries among children up to 17 years old, the risks peak during the first year of life, and overall, the risk is slightly higher for maternal mental illness than paternal mental illness
An increased risk of injuries is most notable for rarer injuries, such as violence related injuries, compared with more common injuries, such as falls
All types of paternal and maternal mental illness show associations with offspring’s risk of injuries and the risk is slightly higher for common mental disorders, such as depression and anxiety, than for more serious mental illness, such as psychosis

## Web extra

Extra material supplied by authors

**Web appendix:** Supplementary files

## Notes

Contributors: AN conceived the study and wrote the protocol. KK, MP, CD, SW, KMA, and MH contributed to the protocol. SW extracted the data. AN performed the data analysis, and AN, KK, MP, CD, KMA, SW, MH, and HH contributed to data interpretation. AN and KK wrote the initial draft and all authors contributed to writing the final manuscript. KMA and CD obtained funding. KK and KMA were cosenior authors of the manuscript. AN and KK are the guarantors of the study. The corresponding author attests that all listed authors meet authorship criteria and that no others meeting the criteria have been omitted.

Ethical approval: The study was approved by the Regional Ethics Review Board in Stockholm, Sweden (Dnr 2010-1185-31-5).

Data sharing: No additional data available.

The manuscript guarantors affirm that this manuscript is an honest, accurate, and transparent account of the study being reported; that no important aspects of the study have been omitted; and that any discrepancies from the study as planned (and, if relevant, registered) have been explained.

Dissemination to participants and related patient and public communities: There are no plans to disseminate the results of the research directly to the study participants. Dissemination to the population, in general, will be through media outreach (eg, press release) on the publication of this study.
