# Supplementary material for: Association between maternal and paternal mental illness and risk of injuries in children and adolescents: nationwide register based cohort study in Sweden
Source: BMJ. 2020 Apr 8;369:m853. doi: 10.1136/bmj.m853 (PMC7190076; doi:10.1136/bmj.m853)
Supplement: Supplementary file 1 — Web appendix: Supplementary files [file neva052515.ww.pdf]

Supplementary Files

Maternal and paternal mental illness and risk of injuries in children and adolescents: a nationwide register-based cohort study in Sweden

Alicia Nevriana (ORCID ID 0000-0003-3434-2757)[1], Matthias Pierce[2], Christina Dalman[1,3], Susanne Wicks[1,3], Marie Hasselberg[1], Holly Hope[2], Kathryn M Abel[2,4], Kyriaki Kosidou[1,3]

Supplementary Figure 1 Exposure and outcome measurements schematic

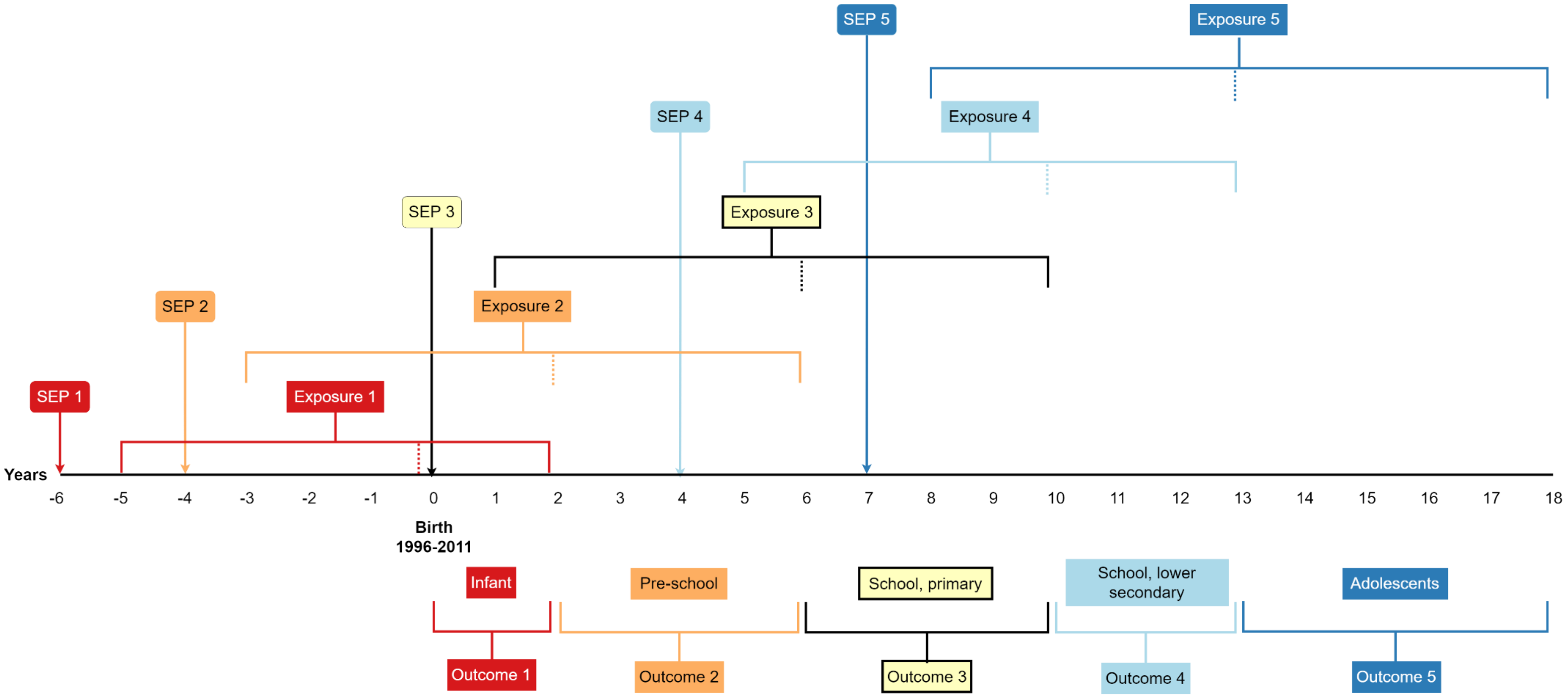

Note:

- Exposure: Parental mental illness
- Outcome: Child injuries
- SEP: Socioeconomic position. Measured at these points: parental education, employment status, family receipt of social welfare benefits, family disposable income in quintiles
- Dotted line: exposure measurement periods for sensitivity analysis

Supplementary Figure 2 Different types of parental mental illness and child's risk of injuries (Adjusted Rate Ratios with 95% CI). Adjusted for sex, birth year, number of siblings (square terms), parental country of birth (missing excluded), maternal age at birth (square terms), paternal age at birth (square terms), living arrangements, parental education, parental employment status, household income.

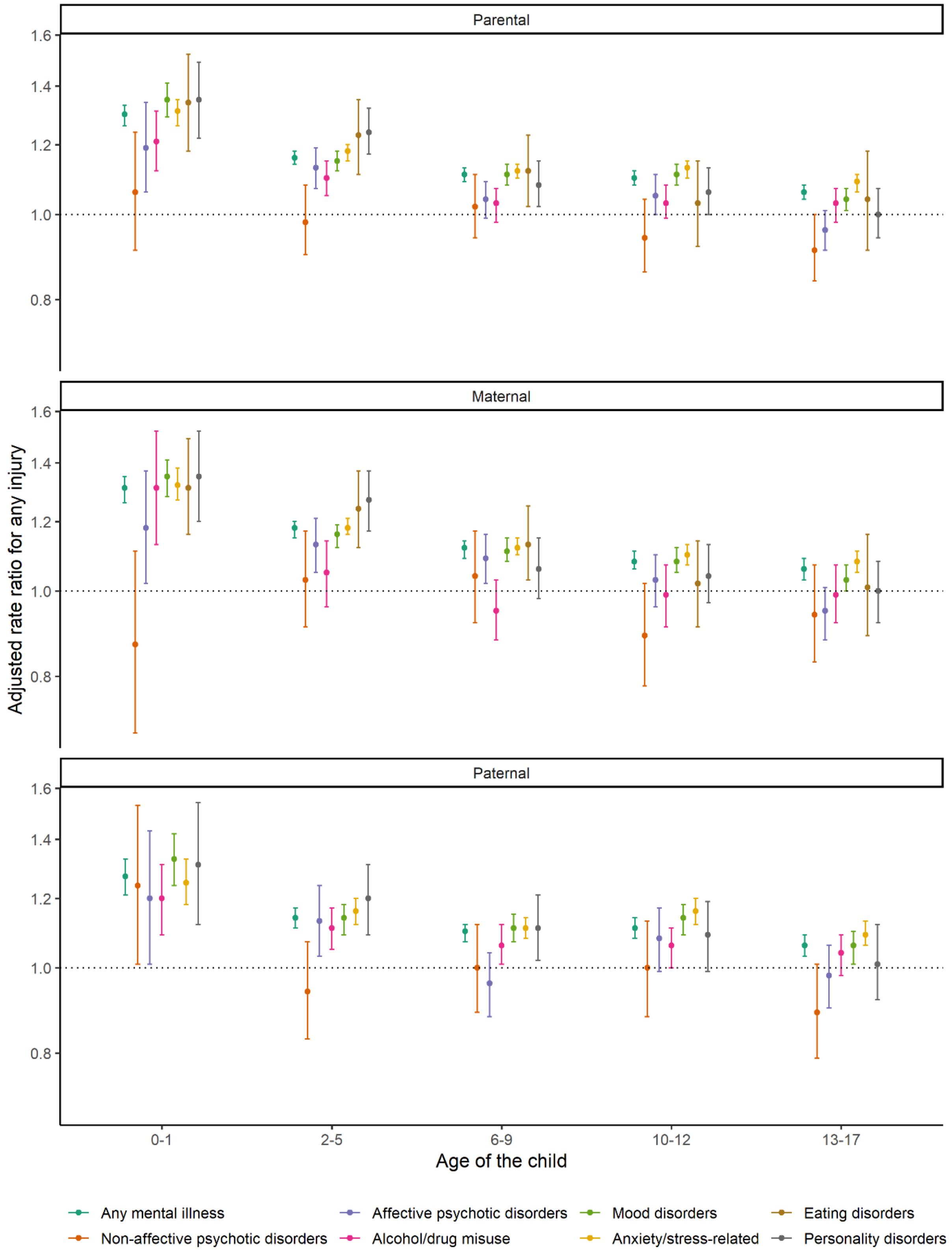

Supplementary Figure 3 Parental mental illness and child's risk of various injuries (Adjusted Rate Ratios with 95% CI). Adjusted for sex, birth year, number of siblings (square terms), parental country of birth (missing excluded), maternal age at birth (square terms), paternal age at birth (square terms), living arrangements, parental education, parental employment status, household income.

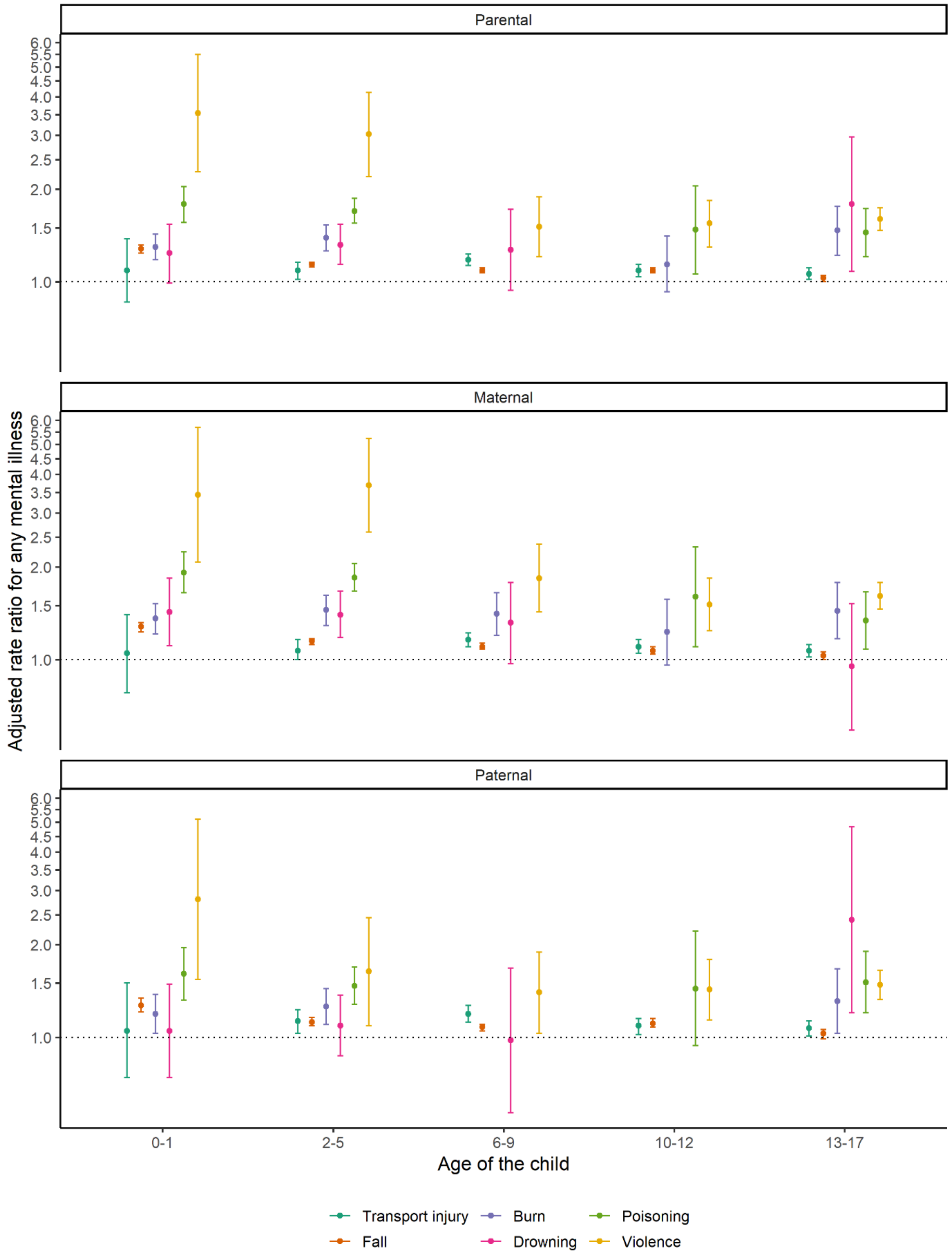

Supplementary Table 1 Distribution of parental socioeconomic position of the children in the study population (N=1,542,000) throughout childhood to adolescence, by the presence of parental mental illness

Variables	Categories	Children (N=1,542,000)									
		Period 1: 0-1 years (n=1,351,683)		Period 2: 2-5 years (n=1,458,868)		Period 3: 6-9 years (n=1,371,158)		Period 4: 10-12 years (n=973,174)		Period 5: 13-17 years (n=700,454)	
		Any parental mental illness		Any parental mental illness		Any parental mental illness		Any parental mental illness		Any parental mental illness	
		CAPRI (N=103,445)	Non-CAPRI (N=1,248,238)	CAPRI (N=181,043)	Non-CAPRI (N=1,277,825)	CAPRI (N=206,425)	Non-CAPRI (N=1,164,733)	CAPRI (N=153,617)	Non-CAPRI (N=819,557)	CAPRI (N=124,655)	Non-CAPRI (N=575,799)
		n (%)	n (%)	n (%)	n (%)	n (%)	n (%)	n (%)	n (%)	n (%)	n (%)
Living arrangement	With neither parent	756 (0.7)	4,917 (0.4)	1,977 (1.1)	2,161 (0.2)	3,059 (1.5)	1,966 (0.2)	2,840 (1.9)	1,729 (0.2)	2,549 (2.0)	1,521 (0.3)
	With one of the parents	22,409 (21.7)	105,004 (8.4)	47,638 (26.3)	124,409 (9.7)	75,220 (36.4)	173,456 (14.9)	67,008 (43.6)	166,404 (20.3)	57,157 (45.9)	134,517 (23.4)
	With both parents	80,280 (77.6)	1,138,317 (91.2)	131,428 (72.6)	1,151,255 (90.1)	128,146 (62.1)	989,311 (84.9)	83,769 (54.5)	651,424 (79.5)	64,949 (52.1)	439,761 (76.4)
Parental highest education	Compulsory	18,373 (17.8)	98,318 (7.9)	27,204 (15.0)	84,095 (6.6)	20,660 (10.0)	49,193 (4.2)	12,189 (7.9)	28,836 (3.5)	9,104 (7.3)	21,137 (3.7)
	Secondary	49,463 (47.8)	591,282 (47.4)	86,872 (48.0)	564,636 (44.2)	102,986 (49.9)	487,176 (41.8)	79,048 (51.5)	346,787 (42.3)	63,230 (50.7)	240,116 (41.7)
	University	30,672 (29.7)	525,653 (42.1)	59,972 (33.1)	588,102 (46.0)	77,971 (37.8)	599,435 (51.5)	59,157 (38.5)	424,071 (51.7)	48,998 (39.3)	296,189 (51.4)
	Missing	4,937 (4.8)	32,985 (2.6)	6,995 (3.9)	40,992 (3.2)	4,808 (2.3)	28,929 (2.5)	3,223 (2.1)	19,863 (2.4)	3,323 (2.7)	18,357 (3.2)
Parental employment status	Unemployed	25,029 (24.2)	154,963 (12.4)	40,207 (22.2)	150,820 (11.8)	35,060 (17.0)	93,582 (8.0)	21,339 (13.9)	46,977 (5.7)	16,367 (13.1)	32,588 (5.7)
	Employed	69,257 (67.0)	848,580 (68.0)	133,096 (73.5)	1,026,785 (80.4)	168,237 (81.5)	1,051,252 (90.3)	130,122 (84.7)	758,331 (92.5)	106,019 (85.1)	529,784 (92.0)
	Missing	9,159 (8.9)	244,695 (19.6)	7,740 (4.3)	100,220 (7.8)	3,128 (1.5)	19,899 (1.7)	2,156 (1.4)	14,249 (1.7)	2,269 (1.8)	13,427 (2.3)
Parental family receipt of social welfare	No	70,186 (67.9)	1,062,519 (85.1)	128,688 (71.1)	1,095,353 (85.7)	159,770 (77.4)	1,048,122 (90.0)	119,797 (78.0)	750,338 (91.6)	98,276 (78.8)	526,822 (91.5)
	Yes	29,815 (28.8)	165,107 (13.2)	48,389 (26.7)	159,428 (12.5)	43,527 (21.1)	96,712 (8.3)	31,664 (20.6)	54,970 (6.7)	24,110 (19.3)	35,550 (6.2)
	Missing	3,444 (3.3)	20,612 (1.7)	3,966 (2.2)	23,044 (1.8)	3,128 (1.5)	19,899 (1.7)	2,156 (1.4)	14,249 (1.7)	2,269 (1.8)	13,427 (2.3)
Parental family disposable income in quintiles	Q1 (lowest)	21,967 (21.2)	210,249 (16.8)	44,167 (24.4)	239,230 (18.7)	33,563 (16.3)	105,110 (9.0)	20,974 (13.7)	54,918 (6.7)	16,141 (13.0)	39,401 (6.8)
	Q2	28,508 (27.6)	382,239 (30.6)	54,321 (30.0)	412,420 (32.3)	54,660 (26.5)	212,584 (18.3)	39,521 (25.7)	124,768 (15.2)	33,128 (26.6)	89,822 (15.6)
	Q3	18,389 (17.8)	221,170 (17.7)	33,038 (18.3)	244,767 (19.2)	62,698 (30.4)	391,375 (33.6)	39,554 (25.8)	214,520 (26.2)	29,378 (23.6)	132,268 (23.0)
	Q4	12,911 (12.5)	160,331 (12.8)	20,950 (11.6)	164,104 (12.8)	32,303 (15.7)	258,905 (22.2)	29,972 (19.5)	223,742 (27.3)	24,393 (19.6)	155,930 (27.1)
	Q5 (highest)	18,228 (17.6)	253,653 (20.3)	24,604 (13.6)	194,272 (15.2)	20,073 (9.7)	176,860 (15.2)	21,440 (14.0)	187,360 (22.9)	19,346 (15.5)	144,951 (25.2)

	Missing	3,442 (3.3)	20,596 (1.7)	3,963 (2.2)	23,032 (1.8)	3,128 (1.5)	19,899 (1.7)	2,156 (1.4)	14,249 (1.7)	2,269 (1.8)	13,427 (2.3)

Supplementary Table 2 Distribution of parental mental illness among children in the study population (N=1,542,000) from childhood through adolescence

Mental illness	Children (N=1,542,000)				
	Period 1: 0-1 years	Period 2: 2-5 years	Period 3: 6-9 years	Period 4: 10-12 years	Period 5: 13-17 years
Parental	N=1,351,683<br>n (%)	N=1,458,868<br>n (%)	N=1,371,158<br>n (%)	N=973,174<br>n (%)	N=700,454<br>n (%)
Any mental illness	103,445 (7.7)	181,043 (12.4)	206,425 (15.1)	153,617 (15.8)	124,655 (17.8)
Non-affective psychotic disorders	4,584 (0.3)	8,125 (0.6)	9,122 (0.7)	6,925 (0.7)	5,743 (0.8)
Affective psychotic disorders	6,772 (0.5)	15,208 (1.0)	20,754 (1.5)	16,923 (1.7)	14,567 (2.1)
Alcohol/drug misuse	16,993 (1.3)	27,944 (1.9)	33,402 (2.4)	26,836 (2.8)	23,732 (3.4)
Mood disorders	42,890 (3.2)	82,062 (5.6)	97,606 (7.1)	73,719 (7.6)	60,478 (8.6)
Anxiety/stress-related	66,220 (4.9)	125,192 (8.6)	146,630 (10.7)	108,104 (11.1)	89,428 (12.8)
Eating disorders	4,456 (0.3)	5,689 (0.4)	5,275 (0.4)	3,410 (0.4)	2,291 (0.3)
Personality disorders	9,448 (0.7)	16,267 (1.1)	18,665 (1.4)	13,853 (1.4)	11,006 (1.6)
Maternal	N=1,350,054<br>n (%)	N=1,456,895<br>n (%)	N=1,368,854<br>n (%)	N=970,751<br>n (%)	N=697,550<br>n (%)
Any mental illness	69,980 (5.2)	120,059 (8.2)	134,523 (9.8)	99,703 (10.3)	81,305 (11.7)
Non-affective psychotic disorders	2,515 (0.2)	4,118 (0.3)	4,451 (0.3)	3,486 (0.4)	2,908 (0.4)
Affective psychotic disorders	4,317 (0.3)	9,691 (0.7)	13,341 (1.0)	10,937 (1.1)	9,377 (1.3)
Alcohol/drug misuse	5,872 (0.4)	8,989 (0.6)	10,681 (0.8)	9,182 (1.0)	8,550 (1.2)
Mood disorders	30,356 (2.3)	55,846 (3.8)	64,505 (4.7)	48,437 (5.0)	39,508 (5.7)
Anxiety/stress-related	45,806 (3.4)	84,693 (5.8)	98,061 (7.2)	72,404 (7.5)	60,587 (8.7)
Eating disorders	4,360 (0.3)	5,523 (0.4)	5,088 (0.4)	3,292 (0.3)	2,204 (0.3)
Personality disorders	6,265 (0.5)	10,310 (0.7)	11,734 (0.9)	8,781 (0.9)	7,000 (1.0)
Paternal	N=1,333,264<br>n (%)	N=1,440,026<br>n (%)	N=1,353,299<br>n (%)	N=956,468<br>n (%)	N=682,002<br>n (%)
Any mental illness	41,975 (3.2)	78,343 (5.4)	92,130 (6.8)	68,435 (7.2)	55,720 (8.2)
Non-affective psychotic disorders	2,128 (0.2)	4,097 (0.3)	4,755 (0.4)	3,500 (0.4)	2,884 (0.4)
Affective psychotic disorders	2,523 (0.2)	5,678 (0.4)	7,624 (0.6)	6,145 (0.6)	5,337 (0.8)
Alcohol/drug misuse	12,511 (0.9)	20,912 (1.5)	24,475 (1.8)	18,919 (2.0)	16,280 (2.4)
Mood disorders	14,229 (1.1)	30,132 (2.1)	37,918 (2.8)	28,809 (3.0)	24,026 (3.5)
Anxiety/stress-related	23,816 (1.8)	48,892 (3.4)	58,596 (4.3)	42,781 (4.5)	35,050 (5.1)
Eating disorders	96 (0.0)	168 (0.0)	188 (0.0)	118 (0.0)	88 (0.0)
Personality disorders	3,419 (0.3)	6,389 (0.4)	7,338 (0.5)	5,327 (0.6)	4,204 (0.6)

Supplementary Table 3 Rate (number of injuries/100,000 person-years) and rate differences of childhood injuries (any type) by age of the child and the type of parental mental illness

Parental mental illness	Children (N=1,542,000)														
	Period 1: 0-1 years (N=1,351,683)			Period 2: 2-5 years (N=1,458,868)			Period 3: 6-9 years (N=1,371,158)			Period 4: 10-12 years (N=973,174)			Period 5: 13-17 years (N=700,454)		
	CAPRI N injuries (Injury rate)	Non-CAPRI N injuries (Injury rate)	Rate difference (95% CI)	CAPRI N injuries (Injury rate)	Non-CAPRI N injuries (Injury rate)	Rate difference (95% CI)	CAPRI N injuries (Injury rate)	Non-CAPRI N injuries (Injury rate)	Rate difference (95% CI)	CAPRI N injuries (Injury rate)	Non-CAPRI N injuries (Injury rate)	Rate difference (95% CI)	CAPRI N injuries (Injury rate)	Non-CAPRI N injuries (Injury rate)	Rate difference (95% CI)
Non-affective psychotic disorders	329 (3,770.8)	82,629 (3,145.3)	625.6 (232.0 to 1,048.4)	1,035 (3,561.4)	192,500 (3,498.9)	62.6 (-150.6 to 284.5)	1,273 (4,145.8)	186,927 (4,112.3)	33.5 (-190.9 to 266.2)	995 (5,630.3)	147,554 (6,065.0)	-434.6 (-778.7 to -76.2)	1,079 (5,285.6)	140,737 (6,081.9)	-796.3 (-1,107.1 to -473.1)
Affective psychotic disorders	628 (4,759.7)	82,330 (3,139.2)	1,620.5 (1,257.2 to 2003.1)	2,529 (4,411.6)	191,006 (3,489.6)	922.0 (751.5 to 1,096.8)	3,154 (4,620.0)	185,046 (4,104.8)	515.1 (354.6 to 679.3)	2,835 (6,667.6)	145,714 (6,051.1)	616.5 (372.1 to 866.9)	2,949 (5,882.7)	138,867 (6,079.1)	-196.4 (-408.6 to 20.8)
Alcohol/drug misuse	1,589 (4,764.3)	81,369 (3,126.6)	1,637.7 (1,406.2 to 1,876.7)	4,481 (4,232.4)	189,054 (3,484.9)	747.5 (623.8 to 873.6)	5,234 (4,641.4)	182,966 (4,099.2)	542.2 (416.2 to 670.5)	4,561 (6,635.5)	143,988 (6,045.3)	590.3 (397.0 to 787.2)	5,599 (6,652.9)	136,217 (6,053.3)	599.6 (423.9 to 778.3)
Mood disorders	4,661 (5,561.9)	78,297 (3,068.0)	2,493.8 (2,334.2 to 2,656.4)	13,853 (4,461.2)	179,682 (3,442.0)	1,019.3 (943.7 to 1,095.6)	15,399 (4,696.5)	172,801 (4,067.5)	629.0 (552.7 to 706.0)	12,781 (6,781.2)	135,768 (6,001.9)	779.3 (658.1 to 901.8)	13,629 (6,368.1)	128,187 (6,045.3)	322.8 (211.4 to 435.2)
Anxiety/stress-related	6,946 (5,386.8)	76,012 (3,032.1)	2,354.7 (2,227.2 to 2,484.2)	21,334 (4,526.6)	172,201 (3,403.5)	1,123.1 (1,060.5 to 1,186.2)	22,894 (4,711.8)	165,306 (4,041.4)	670.4 (606.6 to 734.7)	18,523 (6,785.2)	130,026 (5,971.1)	814.0 (711.5 to 917.4)	20,528 (6,584.0)	121,288 (5,996.4)	587.6 (491.7 to 684.1)
Eating disorders	507 (5,756.5)	82,451 (3,138.6)	2,617.9 (2,130.8 to 3,134.1)	1,054 (4,795.2)	192,481 (3,494.0)	1,301.2 (1,017.1 to 1,597.0)	881 (5,006.1)	187,319 (4,109.1)	897.0 (573.1 to 1,235.4)	623 (7,212.8)	147,926 (6,057.7)	1,155.1 (602.5 to 1,737.2)	557 (7,008.7)	141,259 (6,071.7)	937.0 (370.0 to 1,536.1)
Personality disorders	1,003 (5,398.2)	81,955 (3,131.3)	2,266.9 (1,939.0 to 2,608.6)	2,925 (4,738.1)	190,610 (3,485.2)	1,252.9 (1,082.5 to 1,427.4)	3,080 (4,904.3)	185,120 (4,101.5)	802.7 (630.5 to 979.0)	2,452 (6,900.2)	146,097 (6,049.5)	850.8 (579.4 to 1,129.2)	2,555 (6,615.8)	139,261 (6,065.8)	550.0 (294.7 to 811.8)

CAPRI: Children and Adolescent with PaRental mental Illness

N injuries: Summary number of injuries

Injury rates: Number of injuries/100,000 person-years

Rate difference: Injury rates in exposed-injury rates in unexposed

Supplementary Table 4 Sensitivity analysis on the association between different types of parental mental illness and child's risk of injuries (any type), exposure period 5 years before outcome period e.g. excluding observations exposed at outcome period (Rate Ratio with 95% CI)

Parental mental illness	Children (n=1,542,000)									
	Period 1: 0-1 years		Period 2: 2-5 years		Period 3: 6-9 years		Period 4: 10-12 years		Period 5: 13-17 years	
	N=1,351,683		N=1,458,868		N=1,371,158		N=973,174		N=700,454	
	Any injuries		Any injuries		Any injuries		Any injuries		Any injuries	
	Crude	Adjusted*	Crude	Adjusted*	Crude	Adjusted*	Crude	Adjusted*	Crude	Adjusted*
	Rate Ratio (95% CI)	Rate Ratio (95% CI)	Rate Ratio (95% CI)	Rate Ratio (95% CI)	Rate Ratio (95% CI)	Rate Ratio (95% CI)	Rate Ratio (95% CI)	Rate Ratio (95% CI)	Rate Ratio (95% CI)	Rate Ratio (95% CI)
Any mental illness	1.71 (1.67 to 1.76)	1.28 (1.24 to 1.33)	1.35 (1.32 to 1.37)	1.19 (1.16 to 1.22)	1.18 (1.16 to 1.20)	1.12 (1.09 to 1.14)	1.13 (1.11 to 1.15)	1.11 (1.08 to 1.13)	1.08 (1.06 to 1.10)	1.06 (1.03 to 1.08)
Non-affective psychotic disorders	1.15 (1.00 to 1.33)	1.04 (0.86 to 1.26)	1.06 (0.97 to 1.16)	1.05 (0.94 to 1.19)	1.04 (0.96 to 1.13)	1.05 (0.94 to 1.16)	0.93 (0.85 to 1.02)	0.96 (0.86 to 1.07)	0.85 (0.78 to 0.94)	0.88 (0.80 to 0.98)
Affective psychotic disorders	1.45 (1.31 to 1.61)	1.14 (0.98 to 1.33)	1.27 (1.19 to 1.36)	1.15 (1.06 to 1.26)	1.16 (1.10 to 1.23)	1.07 (0.99 to 1.14)	1.12 (1.06 to 1.18)	1.09 (1.03 to 1.16)	0.95 (0.90 to 1.01)	0.95 (0.89 to 1.01)
Alcohol/drug misuse	1.52 (1.43 to 1.61)	1.20 (1.09 to 1.32)	1.25 (1.20 to 1.31)	1.10 (1.03 to 1.18)	1.17 (1.12 to 1.22)	1.04 (0.98 to 1.10)	1.13 (1.08 to 1.18)	1.06 (1.01 to 1.13)	1.12 (1.07 to 1.17)	1.03 (0.98 to 1.09)
Mood disorders	1.83 (1.76 to 1.90)	1.33 (1.27 to 1.41)	1.35 (1.31 to 1.38)	1.16 (1.12 to 1.21)	1.18 (1.16 to 1.21)	1.12 (1.09 to 1.15)	1.14 (1.11 to 1.17)	1.11 (1.08 to 1.15)	1.06 (1.03 to 1.09)	1.04 (1.01 to 1.08)
Anxiety/stress-related	1.79 (1.74 to 1.85)	1.29 (1.24 to 1.35)	1.39 (1.36 to 1.42)	1.20 (1.17 to 1.24)	1.20 (1.18 to 1.23)	1.14 (1.11 to 1.17)	1.16 (1.13 to 1.18)	1.15 (1.12 to 1.18)	1.11 (1.08 to 1.13)	1.09 (1.06 to 1.13)
Eating disorders	1.77 (1.59 to 1.96)	1.29 (1.12 to 1.49)	1.36 (1.24 to 1.50)	1.17 (1.03 to 1.32)	1.15 (1.04 to 1.27)	1.01 (0.89 to 1.15)	1.20 (1.08 to 1.34)	1.04 (0.91 to 1.19)	1.16 (1.02 to 1.32)	1.02 (0.87 to 1.19)
Personality disorders	1.65 (1.52 to 1.78)	1.22 (1.08 to 1.37)	1.38 (1.30 to 1.46)	1.24 (1.13 to 1.36)	1.23 (1.16 to 1.30)	1.07 (0.99 to 1.16)	1.15 (1.08 to 1.21)	1.04 (0.97 to 1.13)	1.10 (1.04 to 1.17)	1.04 (0.96 to 1.12)

Maternal mental illness	N=1,350,054		N=1,456,895		N=1,368,854		N=970,751		N=697,550	
	Any injuries		Any injuries		Any injuries		Any injuries		Any injuries	
	Crude	Adjusted*	Crude	Adjusted*	Crude	Adjusted*	Crude	Adjusted*	Crude	Adjusted*
	Rate Ratio (95% CI)	Rate Ratio (95% CI)	Rate Ratio (95% CI)	Rate Ratio (95% CI)	Rate Ratio (95% CI)	Rate Ratio (95% CI)	Rate Ratio (95% CI)	Rate Ratio (95% CI)	Rate Ratio (95% CI)	Rate Ratio (95% CI)
Any mental illness	1.77 (1.72 to 1.82)	1.30 (1.25 to 1.35)	1.36 (1.34 to 1.39)	1.19 (1.16 to 1.23)	1.18 (1.16 to 1.21)	1.11 (1.08 to 1.14)	1.12 (1.09 to 1.14)	1.09 (1.06 to 1.12)	1.09 (1.06 to 1.11)	1.06 (1.03 to 1.09)
Non-affective psychotic disorders	1.12 (0.92 to 1.36)	0.91 (0.68 to 1.23)	1.06 (0.94 to 1.21)	1.11 (0.94 to 1.32)	1.02 (0.91 to 1.15)	1.05 (0.91 to 1.22)	0.87 (0.76 to 0.98)	0.90 (0.77 to 1.06)	0.84 (0.73 to 0.96)	0.88 (0.76 to 1.02)
Affective psychotic disorders	1.58 (1.39 to 1.79)	1.16 (0.96 to 1.40)	1.26 (1.15 to 1.37)	1.16 (1.03 to 1.29)	1.18 (1.10 to 1.27)	1.10 (1.01 to 1.21)	1.09 (1.02 to 1.17)	1.06 (0.98 to 1.15)	0.93 (0.86 to 1.00)	0.93 (0.85 to 1.01)

Alcohol/drug misuse	1.67 (1.51 to 1.84)	1.29 (1.09 to 1.53)	1.24 (1.15 to 1.35)	1.02 (0.89 to 1.17)	1.09 (1.01 to 1.18)	0.98 (0.87 to 1.11)	1.05 (0.97 to 1.14)	1.02 (0.92 to 1.12)	1.17 (1.08 to 1.26)	1.08 (0.98 to 1.18)
Mood disorders	1.88 (1.80 to 1.96)	1.36 (1.28 to 1.44)	1.36 (1.32 to 1.41)	1.17 (1.12 to 1.22)	1.19 (1.15 to 1.22)	1.11 (1.07 to 1.15)	1.12 (1.09 to 1.16)	1.09 (1.05 to 1.13)	1.06 (1.03 to 1.10)	1.03 (0.99 to 1.07)
Anxiety/stress-related	1.83 (1.77 to 1.90)	1.32 (1.25 to 1.38)	1.40 (1.36 to 1.43)	1.20 (1.16 to 1.25)	1.20 (1.17 to 1.23)	1.13 (1.10 to 1.17)	1.15 (1.12 to 1.18)	1.12 (1.08 to 1.15)	1.11 (1.08 to 1.14)	1.08 (1.04 to 1.12)
Eating disorders	1.76 (1.58 to 1.95)	1.27 (1.10 to 1.46)	1.36 (1.24 to 1.50)	1.19 (1.04 to 1.35)	1.15 (1.04 to 1.27)	1.02 (0.90 to 1.16)	1.21 (1.09 to 1.35)	1.05 (0.92 to 1.20)	1.15 (1.02 to 1.30)	1.00 (0.86 to 1.16)
Personality disorders	1.71 (1.56 to 1.89)	1.24 (1.07 to 1.44)	1.36 (1.26 to 1.47)	1.25 (1.11 to 1.40)	1.23 (1.15 to 1.33)	1.06 (0.96 to 1.18)	1.14 (1.06 to 1.23)	1.03 (0.93 to 1.13)	1.09 (1.02 to 1.18)	1.03 (0.93 to 1.13)

Paternal mental illness	N=1,333,264		N=1,440,026		N=1,353,299		N=956,468		N=682,002	
	Any injuries		Any injuries		Any injuries		Any injuries		Any injuries	
	Crude	Adjusted*	Crude	Adjusted*	Crude	Adjusted*	Crude	Adjusted*	Crude	Adjusted*
	Rate Ratio (95% CI)	Rate Ratio (95% CI)	Rate Ratio (95% CI)	Rate Ratio (95% CI)	Rate Ratio (95% CI)	Rate Ratio (95% CI)	Rate Ratio (95% CI)	Rate Ratio (95% CI)	Rate Ratio (95% CI)	Rate Ratio (95% CI)
Any mental illness	1.58 (1.52 to 1.64)	1.24 (1.17 to 1.30)	1.30 (1.26 to 1.34)	1.17 (1.13 to 1.21)	1.16 (1.14 to 1.19)	1.11 (1.08 to 1.14)	1.13 (1.10 to 1.16)	1.13 (1.09 to 1.16)	1.06 (1.03 to 1.09)	1.07 (1.03 to 1.10)
Non-affective psychotic disorders	1.18 (0.96 to 1.45)	1.15 (0.89 to 1.49)	1.05 (0.92 to 1.19)	1.00 (0.85 to 1.18)	1.05 (0.94 to 1.19)	1.04 (0.90 to 1.20)	0.99 (0.88 to 1.12)	1.01 (0.88 to 1.17)	0.87 (0.77 to 0.98)	0.90 (0.77 to 1.04)
Affective psychotic disorders	1.26 (1.05 to 1.51)	1.11 (0.88 to 1.41)	1.29 (1.17 to 1.44)	1.15 (1.00 to 1.31)	1.10 (1.00 to 1.21)	1.00 (0.89 to 1.12)	1.15 (1.05 to 1.25)	1.13 (1.02 to 1.24)	1.00 (0.91 to 1.10)	0.99 (0.89 to 1.10)
Alcohol/drug misuse	1.44 (1.34 to 1.55)	1.18 (1.06 to 1.32)	1.26 (1.19 to 1.32)	1.14 (1.06 to 1.22)	1.19 (1.14 to 1.25)	1.06 (0.99 to 1.13)	1.15 (1.09 to 1.21)	1.08 (1.01 to 1.16)	1.08 (1.02 to 1.14)	1.01 (0.95 to 1.08)
Mood disorders	1.71 (1.60 to 1.83)	1.27 (1.16 to 1.39)	1.28 (1.22 to 1.34)	1.15 (1.08 to 1.22)	1.18 (1.13 to 1.22)	1.13 (1.08 to 1.19)	1.16 (1.11 to 1.21)	1.15 (1.10 to 1.21)	1.06 (1.01 to 1.11)	1.08 (1.03 to 1.14)
Anxiety/stress-related	1.66 (1.58 to 1.75)	1.22 (1.14 to 1.31)	1.36 (1.32 to 1.41)	1.20 (1.14 to 1.25)	1.19 (1.15 to 1.23)	1.14 (1.10 to 1.19)	1.16 (1.12 to 1.20)	1.19 (1.14 to 1.23)	1.09 (1.05 to 1.13)	1.11 (1.07 to 1.16)
Personality disorders	1.49 (1.29 to 1.71)	1.16 (0.95 to 1.42)	1.37 (1.24 to 1.51)	1.20 (1.05 to 1.39)	1.24 (1.14 to 1.35)	1.08 (0.96 to 1.21)	1.14 (1.05 to 1.25)	1.07 (0.95 to 1.20)	1.11 (1.01 to 1.22)	1.06 (0.94 to 1.20)

*Adjusted for sex, birth year, number of siblings (square terms), parental country of birth (missing excluded), maternal age at birth (square terms), paternal age at birth (square terms), living arrangements, parental education, parental employment status, household income.

Analyses for paternal eating disorders were not conducted since there were very few observations.

Supplementary Table 5 Sensitivity analysis on the association between different types of parental mental illness and child's risk of injuries (any type) during age 0-1 years, including only children known to be living with at least one of their birth parents

Type of mental illness	Children (n=1,542,000)					
	Period 1: 0-1 years					
	Parental mental illness		Maternal mental illness		Paternal mental illness	
	N children=1,346,010		N children=1,344,406		N children=1,328,021	
	Any injuries		Any injuries		Any injuries	
	Crude	Adjusted*	Crude	Adjusted*	Crude	Adjusted*
	Rate Ratio (95% CI)	Rate Ratio (95% CI)	Rate Ratio (95% CI)	Rate Ratio (95% CI)	Rate Ratio (95% CI)	Rate Ratio (95% CI)
Any mental illness	1.70 (1.66 to 1.73)	1.30 (1.26 to 1.34)	1.75 (1.70 to 1.79)	1.31 (1.26 to 1.35)	1.61 (1.56 to 1.67)	1.27 (1.21 to 1.33)
Non-affective psychotic disorders	1.20 (1.07 to 1.35)	1.07 (0.91 to 1.25)	1.12 (0.96 to 1.32)	0.88 (0.69 to 1.12)	1.29 (1.10 to 1.52)	1.25 (1.02 to 1.53)
Affective psychotic disorders	1.51 (1.39 to 1.64)	1.18 (1.05 to 1.32)	1.59 (1.44 to 1.76)	1.16 (1.00 to 1.35)	1.40 (1.22 to 1.60)	1.20 (1.01 to 1.43)
Alcohol/drug misuse	1.52 (1.44 to 1.61)	1.22 (1.12 to 1.32)	1.71 (1.56 to 1.87)	1.32 (1.14 to 1.53)	1.46 (1.38 to 1.56)	1.20 (1.09 to 1.31)
Mood disorders	1.81 (1.75 to 1.87)	1.35 (1.29 to 1.41)	1.83 (1.77 to 1.90)	1.35 (1.28 to 1.42)	1.74 (1.65 to 1.84)	1.33 (1.23 to 1.42)
Anxiety/stress-related	1.77 (1.73 to 1.82)	1.31 (1.26 to 1.35)	1.80 (1.75 to 1.86)	1.32 (1.27 to 1.38)	1.70 (1.62 to 1.77)	1.25 (1.19 to 1.33)
Eating disorders	1.81 (1.65 to 1.99)	1.32 (1.16 to 1.49)	1.80 (1.63 to 1.98)	1.29 (1.14 to 1.47)	2.19 (1.17 to 4.08)	2.53 (1.23 to 5.19)
Personality disorders	1.72 (1.61 to 1.84)	1.34 (1.22 to 1.48)	1.77 (1.63 to 1.92)	1.34 (1.19 to 1.51)	1.60 (1.43 to 1.79)	1.32 (1.12 to 1.55)

*Adjusted for sex, birth year, number of siblings (square terms), parental country of birth (missing excluded), maternal age at birth (square terms), paternal age at birth (square terms), living arrangements, parental education, parental employment status, household income.

Supplementary Table 6 ICD codes used for identifying childhood psychopathology in the National Patient Register

Childhood psychopathology diagnoses	ICD-9 codes	ICD-10 codes
Psychotic disorders	291-298	F20-29
Alcohol/drug misuse	303-305	F10-F19
Mood disorders	311	F30-F39
Anxiety and stress-related disorders	300, 306, 308-309	F40-F48
Behavioural syndromes associated with physiological disturbances and physical factors	302, 316	F50-F59
Personality disorders	301, 312	F60-F59
Pervasive developmental disorders	299	F84
Child- and adolescent-onset behavioural and emotional disorders	307, 313, 314	F90-95
